# The role of autophagy in the process of osseointegration around titanium implants with micro-nano topography promoted by osteoimmunity

**DOI:** 10.1038/s41598-021-98007-7

**Published:** 2021-09-16

**Authors:** Ting Zhang, Mengyang Jiang, Xiaojie Yin, Peng Yao, Huiqiang Sun

**Affiliations:** 1 Department of Prosthodontics, School and Hospital of Stomatology, Cheeloo College of Medicine, Shandong University and Shandong Key Laboratory of Oral Tissue Regeneration and Shandong Engineering Laboratory for Dental Materials and Oral Tissue Regeneration, Jinan, Shandong China; 2 School of Mechanical Engineering, Shandong University, Jinan, Shandong China

**Keywords:** Implants, Autophagy, Osteoimmunology

## Abstract

Osteoimmunity plays an important role in the process of implant osseointegration. Autophagy is a conservative metabolic pathway of eukaryotic cells, but whether the interaction between autophagy and osteoimmunity plays a key role in osseointegration remains unclear. In this study, we prepared smooth titanium disks and micro-nano topography titanium disks, to study the immune microenvironment of RAW264.7 cells, and prepared the conditioned medium to study the effect of immune microenvironment on the osteogenesis and autophagy of MC3T3-E1 cells. Autophagy inhibitor 3-MA was used to inhibit autophagy to observe the change of expression of osteogenic markers. The results showed that the micro-nano topography titanium disks could stimulate RAW264.7 cells to differentiate into M2 type, forming an anti-inflammatory immune microenvironment; compared with the control group, the anti-inflammatory immune microenvironment promoted the proliferation and differentiation of osteoblasts better. The anti-inflammatory immune environment activated the autophagy level of osteoblasts, while the expression of osteogenic markers was down-regulated after inhibition of autophagy. These results indicate that anti-inflammatory immune microenvironment can promote cell proliferation and osteogenic differentiation, autophagy plays an important role in this process. This study further explains the mechanism of implant osseointegration in osteoimmune microenvironment, and provides reference for improving implant osseointegration.

## Introduction

With the development of dental implant technology and bone biomaterials, implant denture has become the preferred treatment for dentition defect and complete edentulism. Rapid and effective osseointegration is the key to implant success^1^. The successful osseointegration benefits from the surface modification of implants^2^, such as Sandblasted, Large-grit, Acid-etched (SLA) technology, laser treatment, anodizing, alkali-heat treatment^3–5^, etc. SLA technology is the most widely used surface modification method in commercial implants at this stage. It increases the surface area of implants at the micron level and promotes bone integration at the cellular level^2,6^. In recent years, it has been found that nano topography can increase the surface energy of implants^7^, affect the interaction between implant and bone interface from the level of cells and proteins^8^, promote osteoblast adhesion^9^, and thus potentially promote osseointegration. The micro-nano topography can simultaneously combine the advantages of micron topography and nano topography. Compared with the traditional SLA micron topography, the micro-nano topography has a stronger ability to form hydroxyapatite in vitro, which can better promote the adhesion and extension of bone cells, and promote the osseointegration of implants^10–12^. In our previous study, we found that compared with smooth, micron topography and nano topography, the micro-nano topography titanium disk obtained by SLA technology combined with alkali-heat treatment promoted the proliferation and differentiation of MC3T3-E1 cells better^13^.

In recent years, with the progress of immunology and deep understanding of bone remodeling, a new term—"Osteoimmunity" has emerged, which indicates the close relationship between immune system and skeletal system. The immune system can affect tissue repair and regeneration, and is an effective method for inducing tissue regeneration^14^, including bone regeneration^15^. Macrophages are highly heterogeneous immune cells derived from the myeloid system. Their interaction with bone cells is crucial for bone homeostasis^16^. Tissue damage caused by external (injury, chemical, infection, etc.) and internal factors (DNA damage, immune response, etc.) can induce macrophage activation, activate specific transcription programs, transform into pro-inflammatory M1 and/or anti-inflammatory M2 phenotypes, release pro-inflammatory and/or anti-inflammatory mediators into microenvironment, regulate immune response, and affect bone homeostasis^17,18^. Many studies have shown that immune cell response is affected by various factors, including surface topography^19,20^, chemical components^21,22^, porosity^23^, stiffness^24–26^, etc. Macrophages have strong plasticity, in which the surface morphology plays a leading role in regulating immune response^27^. Some studies have shown that macrophages have different responses to different surface roughness and topography. Luu et al.^27^ showed that both micron and nano grooved titanium disks could affect macrophage elongation and induce macrophages to polarize into M2 phenotype with anti-inflammatory and healing promoting effects. The results of Pan et al.^28^ showed that the micro-nano topography obtained by plasma-sprayed and alkaline thermal treatment could induce stronger cytoskeleton tension than the micron topography obtained by plasma-sprayed treatment, which made macrophages polarized to M2 type and regulated the expression of inflammatory genes, and the gene expression of BMP-2 and VEGF of bone marrow mesenchymal stem cells (BMSCs) was also up-regulated. In our previous study, we found that micro-nano topography titanium disks could induce RAW264.7 cells to polarize into anti-inflammatory M2 phenotype, and promote the migration, proliferation and osteogenic differentiation of MC3T3-E1 cells^13^.

Autophagy is a conserved and important cellular metabolic pathway in mammalian cells. Under starvation, hypoxia and oxidative stress, autophagosomes can capture damaged macromolecules and organelles, degrade them by fusing with lysosomes and recycle them to ensure cell survival and growth^29,30^. Autophagy plays a key role in maintaining homeostasis in vivo^31^, is one of the most basic responses of cells to adapt to the new microenvironment^32^. Recent studies have shown that autophagy plays a key role in bone remodeling^33–35^. Cheng et al.^35^ found that strontium induced osteogenic differentiation of MC3T3-E1 cells depended on autophagy. After treatment with autophagy inhibitor 3-mA, the osteogenic differentiation effect of MC3T3-E1 cells induced by strontium was down-regulated, which indicated that autophagy was important in the process of osteogenic differentiation of MC3T3-E1 cells. Kalu et al.^32^ confirmed that rough titanium disk surface can promote the proliferation, maturation and differentiation of osteoblasts, but after using autophagy inhibitor 3-MA, the proliferation, maturation and differentiation of osteoblasts are inhibited, thus inhibiting the osseointegration of implants. This indicates that autophagy plays an important role in the osseointegration of rough titanium surface.

However, whether autophagy is involved in the regulation of implant osseointegration in bone immune microenvironment is unknown. Therefore, this study focused on the mechanism of implant osseointegration in bone immune microenvironment, explored whether autophagy participated in this process, and further studied the related mechanisms of implant osseointegration.

## Results

### Surface characterization of titanium disk

**Figure 1:**
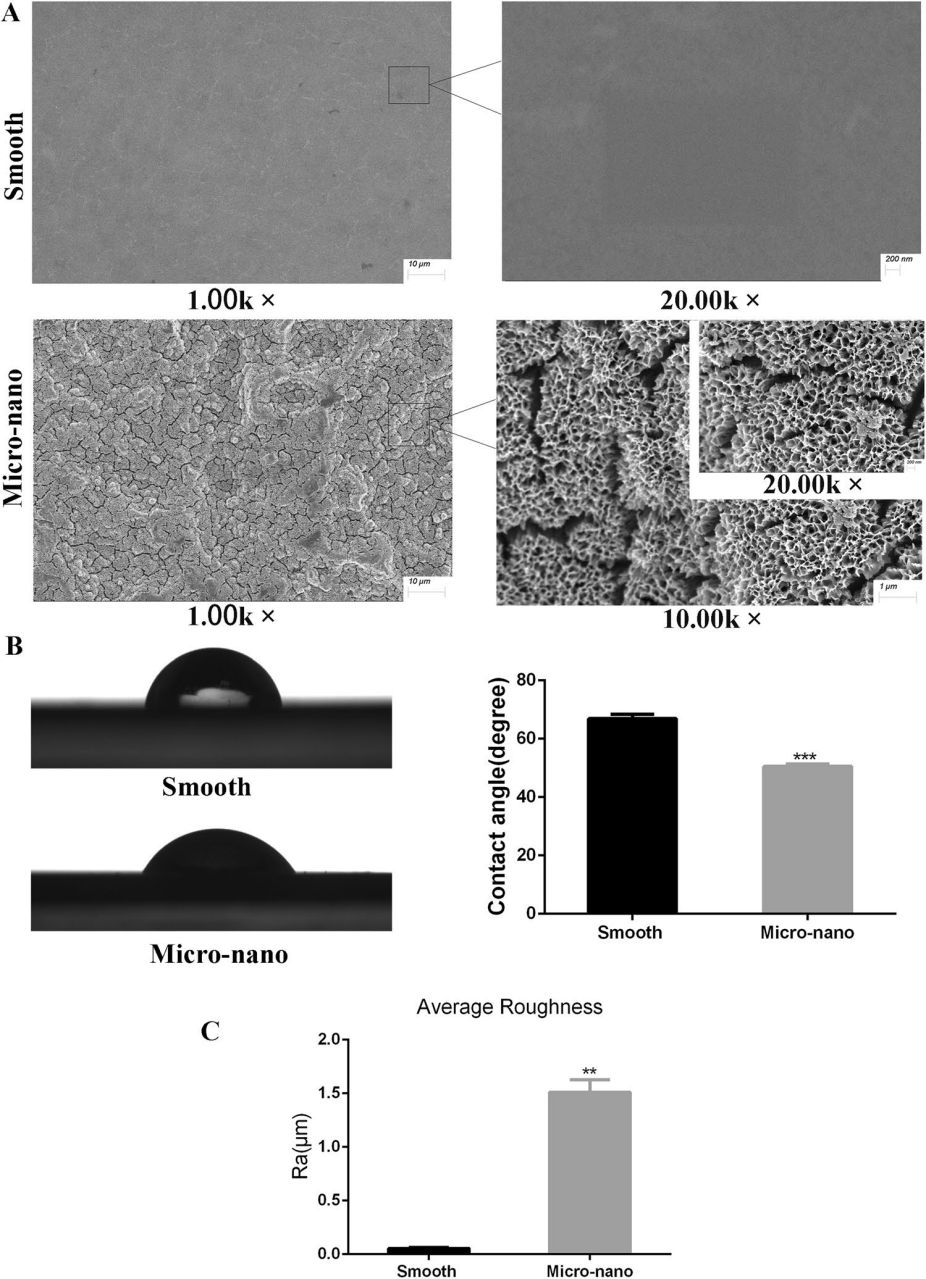
Characterization of material surface. (**A**) Scanning electron of surface topography. The smooth titanium disk was at ×1.00kand ×20.00k magnification; and the micro-nano titanium disk was at ×1.00k, ×10.00k and ×20.00k magnification. (**B**) Contact angle test. (**C**) Roughness detection (*smooth* smooth titanium disk, *Micro-nano* micro-nano topography titanium disk. *p < 0.05; **p < 0.01; ***p < 0.001).

After ultrasonic cleaning and drying at room temperature, the surfaces of the two kinds of titanium disks were sprayed with gold, and the scanning electron microscope (SEM) observation was showed in Fig. 1A. The surface of the smooth titanium disks was smooth and no obvious scratch was found; under the low power microscope (1.00k×), the surface of the micro-nano topography titanium disks was rough, no residual alumina particles were found, and a large number of pits were visible. The pits were in the shape of multi-stage continuous superposition, and on the surface of the first-order depression with a diameter of about 10–50 μ m, a secondary depression with a diameter of 2–8 μ m is superimposed (10.00k×). The nano pores with a diameter of 50–200 nm can be seen under high power microscope (20.00k×).

Figure 1B shows the droplet morphology and average surface hydrophilicity of the two kinds of titanium disks. When 2ul artificial saliva was used as wetting agent, the contact angle of micro-nano topography titanium disk was significantly lower than that of smooth titanium disk (*p* < 0.05). Optical profilometer was used to observe and compare the average surface roughness Ra of the two kinds of titanium disks. The results are shown in Fig. 1C. Compared with smooth titanium disks, the average surface roughness of micro-nano topography titanium disks increased significantly (*p* < 0.05), which was consistent with the results of SEM.

### Polarization of macrophages to anti-inflammatory M2 induced by micro-nano topography titanium disk

**Figure 2:**
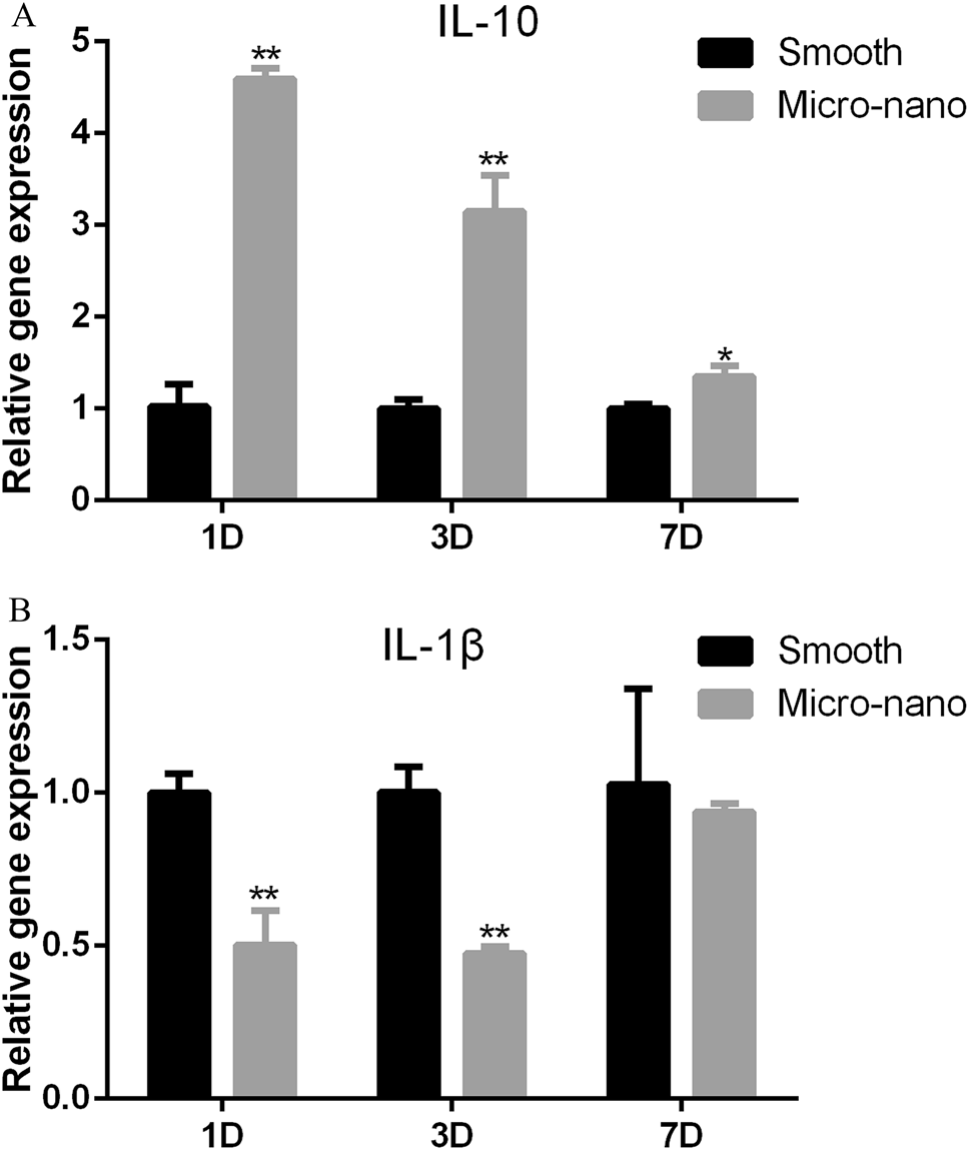
Gene expression of RAW264.7 cells on different titanium disks. (**A**) IL-10; (**B**) IL-1β (*smooth* smooth titanium disk, *Micro-nano* micro-nano topography titanium DISK. *p < 0.05; **p < 0.01).

After RAW264.7 cells were cultured on the surface of smooth, micro-nano topography titanium disk, the expression of inflammatory related genes was detected. Compared with smooth titanium disk, the expression of anti-inflammatory gene IL-10 on RAW264.7 cells with micro-nano topography increased, and the expression of pro-inflammatory related factor IL-1 β was decreased (Fig. 2), which was consistent with our previous research results^13^.

### The effect of bone immunity on MC3T3-E1 cells proliferation and differentiation

**Figure 3:**
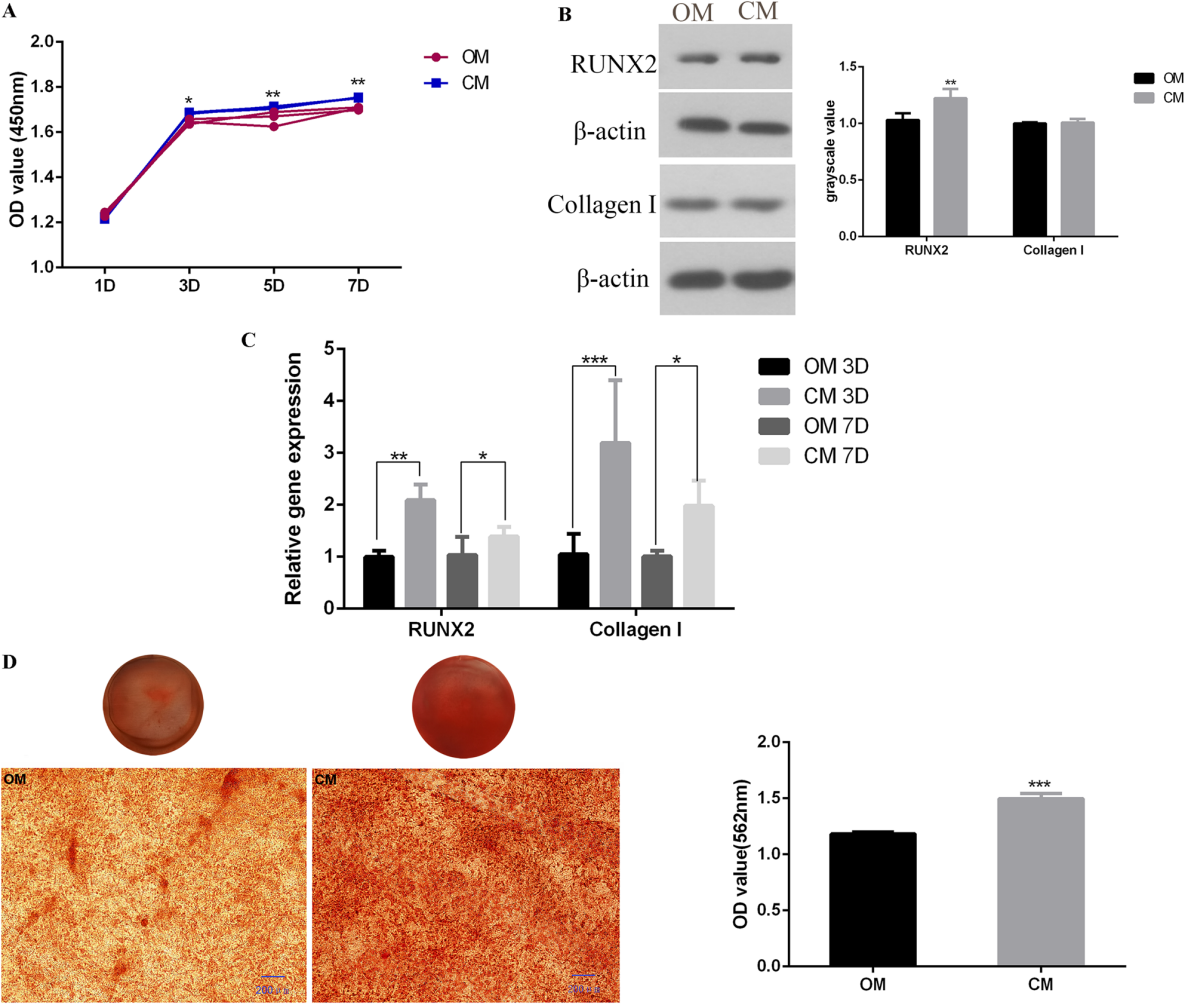
Comparison of the proliferation, differentiation and mineralization levels of osteoblasts stimulated with or without macrophage medium supernatant. (**A**) CCK-8 was used to detect the proliferation activity of MC3T3-E1 cells at 1, 3, 5, 7 days; (**B**) western blot showed the expression of osteogenic related proteins runx2 and collagen I at 7 days; (**C**) real-time PCR showed the expression of osteogenic marker genes *runx2* and collagen I at 3 and 7 days; (**D**) alizarin red showed the mineralization of MC3T3-E1 cells [*OM* osteogenic induction medium, *CM* conditional medium (osteogenic induction medium and the supernatant of RAW264.7 cells’ culture medium). *p < 0.05; **p < 0.01; ***p < 0.001].

In order to study the effect of OM and CM on the activity of MC3T3-E1 cells, CCK-8 was used to study cell proliferation (Fig. 3A). The results showed that OM and CM had no obvious cytotoxicity, and compared with OM, CM can promote MC3T3-E1 cell proliferation better. In order to detect the osteogenic differentiation of MC3T3-E1 cells under different treatments, qRT-PCR and Western blot were used to detect the expression of osteogenic marker genes (Fig. 3B,C, Supplementary file 1^36^). The results which had been shown in our previous research^36^, showed that CM significantly enhanced the mRNA expression of runx2 and collagen I in MC3T3-E1 cells compared with OM. In terms of protein expression, compared with OM, CM enhanced the protein expression of runx2, and the difference was statistically significant (*p* < 0.05). CM also enhanced the protein expression of collagen I, but the difference is not statistically significant. In order to explore the effect of osteoimmune microenvironment on extracellular matrix mineralization of MC3T3-E1, alizarin red staining was performed on the cells. Microscopic and semi quantitative results showed that compared with the control group, the mineral deposition of cells in the osteoimmune microenvironment was increased (Fig. 3D).

### Bone immune microenvironment affects autophagy of MC3T3-E1 cells

**Figure 4:**
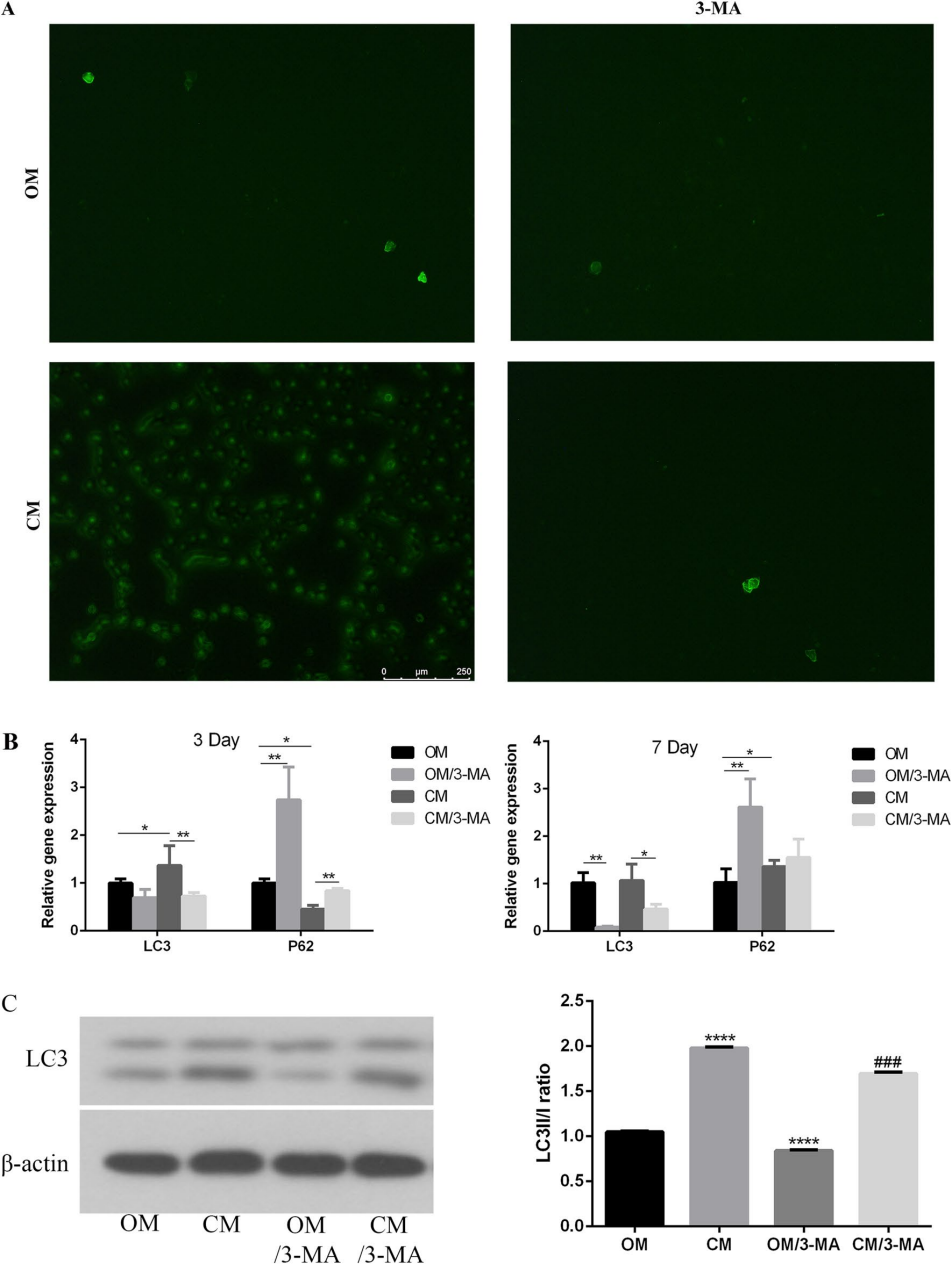
Detection of autophagy level of MC3T3-E1 cells under OM and CM culture conditions with or without 3-MA. (**A**) MDC autophagy body staining (Autophagosomes were shown in green); (**B**) real-time quantitative PCR showed the expression of autophagy related genes LC3 and P62 at day 3 and 7. (**C**) Western blot showed the expression of LC3II/[*OM* osteogenic induction medium, *OM/3-MA* adding 5 mM autophagy inhibitor 3-MA to osteogenic induction medium, *CM* conditional medium (osteogenic induction medium and the supernatant of RAW264.7 cells’ culture medium), *CM/3-MA* adding 5 mM autophagy inhibitor 3-MA to the conditioned medium. *p < 0.05; **p < 0.01; ***p < 0.001; ****p < 0.0001].

In order to explore whether autophagy is involved in the process of osteoimmune microenvironment affecting the osteogenesis of MC3T3-E1 cells, the autophagic vesicles were observed by MDC staining. The results showed that compared with the control group, the number of autophagic vesicles in osteoimmune microenvironment was significantly increased, and which decreased after using autophagy inhibitor 3-MA (Fig. 4A). The expression of autophagy related genes was detected by qRT-PCR. The results showed that compared with the control group, the expression of LC3 and p62 in osteoimmune microenvironment increased on day 3 and day 7, and the expression level of LC3 and p62 decreased after using autophagy inhibitor 3-MA (Fig. 4B). At the same time, Western blot results showed that CM increased the LC3II/I ratio of MC3T3-E1 cells, and which decreased with the addition of autophagy inhibitor 3-MA, but it was slightly higher than that of 3-MA alone (Fig. 4C, Supplementary file 1). These results indicate that osteoimmune microenvironment can activate autophagy of MC3T3-E1 cells.

### MC3T3-E1 cells proliferation and differentiation activity after autophagy inhibition

**Figure 5:**
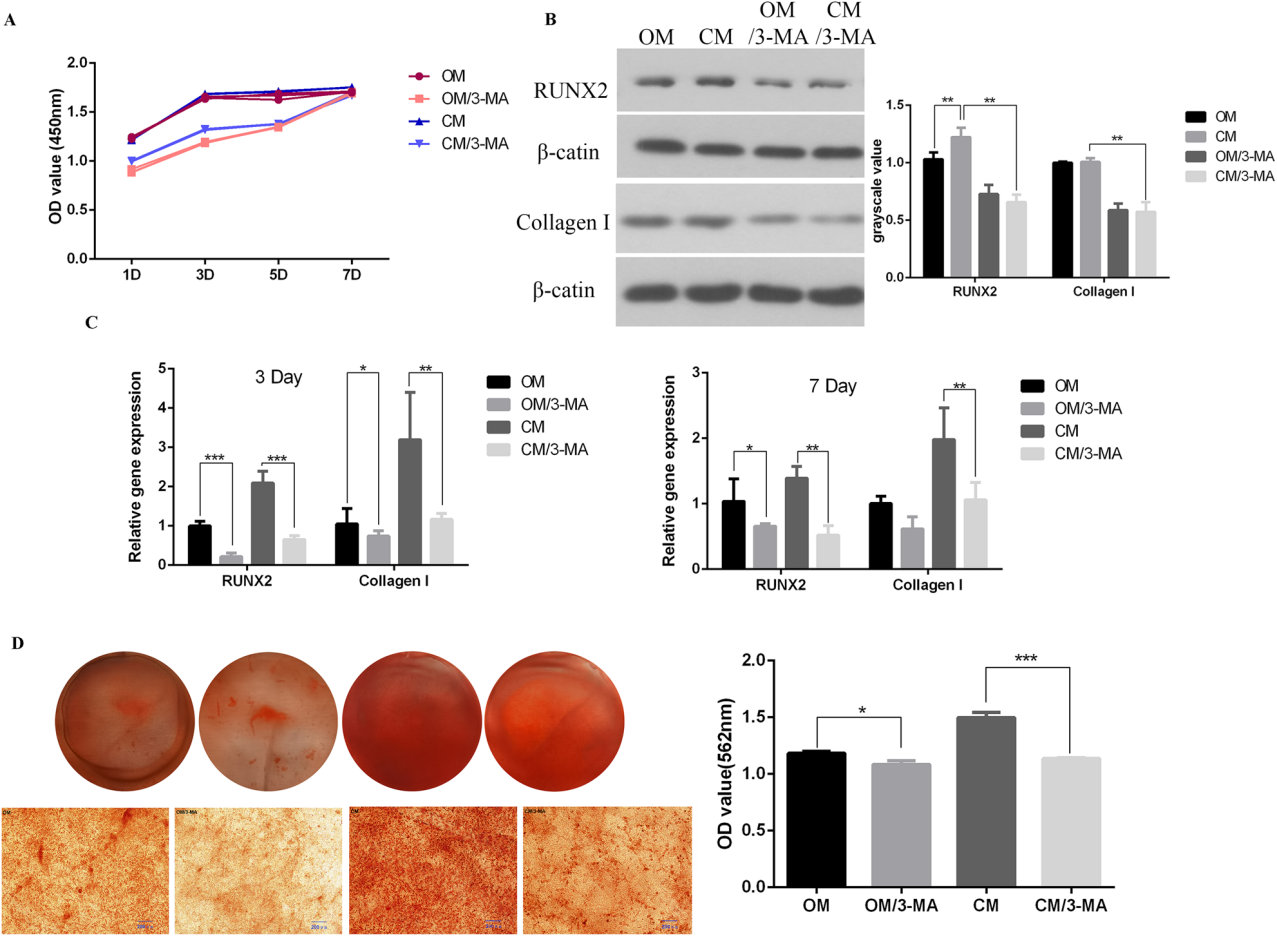
Comparison of the proliferation, differentiation and mineralization levels of MC3T3-E1 cells in OM and CM groups with or without 3-MA pretreatment. (**A**) CCK-8 was used to detect the proliferation activity of MC3T3-E1 cells at 1, 3, 5, 7 days; (**B**) western blot showed the expression of osteogenic related proteins runx2 and collagen I at 7 days; (**C**) real-time PCR showed the expression of osteogenic marker genes *runx2* and collagen I at 3 and 7 days; (**D**) alizarin red showed the mineralization of MC3T3-E1 cells [*OM* osteogenic induction medium, *OM/3-MA* adding 5 mM autophagy inhibitor 3-MA to osteogenic induction medium, *CM* conditional medium (osteogenic induction medium and the supernatant of RAW264.7 cells’ culture medium), *CM/3-MA* adding 5 mM autophagy inhibitor 3-MA (*p < 0.05; **p < 0.01; **p < 0.001; **p < 0.0001)].

In order to detect the effect of autophagy on MC3T3-E1 cells in osteoimmune environment, CCK-8 was used to study cell proliferation (Fig. 5A). The results showed that the proliferation of MC3T3-E1 cells was significantly reduced by adding autophagy inhibitor 3-MA to OM and CM media, but gradually returned to the level close to that of the control group over time, which indicated that autophagy could affect the proliferation of MC3T3-E1 cells. After autophagy inhibitor (3-MA) was used to inhibit autophagy, qRT-PCR and Western blot were used to detect the expression of osteogenesis related marker genes and proteins (Fig. 5B,C, Supplementary file^36^). The results showed that compared with the group without 3-MA, the expression of runx2 and Collagen I decreased. In order to explore the effect of autophagy on extracellular matrix mineralization of MC3T3-E1, alizarin red staining was performed on the cells. Microscopic and semi quantitative results showed that compared with the group without 3-mA, the mineral deposition of MC3T3-E1 cells was decreased after autophagy inhibition (Fig. 5D).

## Discussion

In the process of implant osseointegration, the communication between cells and cells, cells and materials are indispensable, especially in the early stage^2^. In this study, we investigated the biological behavior of macrophage RAW264.7 on MC3T3-E1 cells in osteoimmune microenvironment, and explored the key role of autophagy in osteoimmune regulation of bone integration. The main finding is that autophagy can effectively regulate the osseointegration of implants in osteoimmune microenvironment.

Surface modification of implants is very important for successful implant osseointegration. As mentioned above^13^, we used SLA technology combined with alkali-heat treatment to prepare micro-nano topography titanium disk, which has the basic surface morphology of SLA titanium disk, that is, the primary sand blasting depression with diameter of 10–50 μ m is superimposed with secondary acid etching depression with diameter of 2–8 μM, and nano pore with diameter of about 50–200 nm is contained at the same time. In this way, the surface roughness of the micro-nano topography is significantly improved, and it is more hydrophilic. Moreover, we have confirmed that the proliferation and differentiation of MC3T3-E1 cells on the surface of micro-nano topography titanium disks are better than those of smooth, micron and nano titanium disks, and the effect of promoting bone integration is better^13^.

Implant osseointegration is driven by inflammatory process^37^. After the initial implantation of implants, the host tissue damage leads to blood extravasation, which activates the host's immune response, namely osteoimmunity. Macrophages play an important role in osteoimmunity. Macrophage is an important part of innate immunity, which has heterogeneity and plasticity, and plays a key role in maintaining homeostasis and immune regulation^38^. After implantation, the first immune component contacting the implant is macrophage, which can secrete a variety of cytokines and mediators, including proinflammatory cytokines (such as tumor necrosis factor alpha), anti-inflammatory mediators (such as interleukin-10) and growth factors (such as transforming growth factor beta)^39^, which regulate the host immune response, affect the material cell interface reaction between the implant and host cells, and moderate inflammation. Moderate inflammation-related cytokines can influence osteogenic differentiation of mesenchymal stem cells, and play an important role in bone formation and bone remodeling, and influence the osseointegration of implants^27,40^, while excessive inflammation is not conducive to osseointegration. Therefore, planting materials with immunomodulatory function is another concern. It has been confirmed in our previous experiments that micro-nano topography titanium disk can induce RAW264.7 cells to polarize to M2 type, secrete anti-inflammatory mediators IL-10, etc., to form an anti-inflammatory osteoimmune microenvironment, and then promote the migration of osteoblasts^13^. In this study, the supernatant of RAW264.7 cells’ culture medium on the surface of micro-nano topography titanium disk was collected and added to the osteoblast culture medium. After the osteoblast culture environment was adjusted to anti-inflammatory osteoimmune microenvironment, the gene and protein expression of runx2 and collagen I of MC3T3-E1 cells were higher than those of the control group, that is, osteoimmune microenvironment had better effect on promoting osteogenic differentiation.

Autophagy is a self-degradation process in eukaryotic cells, which can be used as a dynamic circulatory system for cell regeneration and homeostasis^41^. Autophagy has been shown to play an important role in the process of osteogenic differentiation^42–44^. Autophagy plays an important role in the mineralization of osteoblasts during bone formation. Autophagy can be used as a carrier to transport mineralization related factors to the extracellular matrix in the form of stromal vesicles, and then participate in the regulation of osteoblast mineralization^45^. Microtubule associated protein 1A/1B-light chain 3 (LC3) is a soluble protein. During autophagy, cytoplasmic LC3 (LC3-I) is coupled with phosphatidylethanolamine to form LC3 phosphatidylethanolamine coupling compound (LC3-II), which is raised on autophagy membrane. Therefore, LC3-II/I is considered as the marker of autophagy^46^. Autophagy has been shown to play an important role in bone homeostasis. Lu et al.^47^ confirmed that iRoot BP plus can promote bone/odontogenic differentiation of mesenchymal stem cells through MAPK pathway and autophagy. The study by Kaluđerović et al.^32^ confirmed that rough titanium surface could promote the proliferation, maturation and differentiation of osteoblasts by stimulating autophagy dependent PI3/Akt signal transduction pathway. In our study, MC3T3-E1 cells were stimulated by the supernatant of RAW264.7 cells’ medium cultured on micro-nano topography titanium disks, resulting in the increase of LC3II/I ratio. Meanwhile, the number of autophagic vesicles in MC3T3-E1 cells was significantly increased by MDC staining, indicating that autophagy was activated. In order to further prove that autophagy plays a key role in the process of osteoimmune microenvironment promoting bone integration, we used autophagy inhibitor 3-MA to inhibit the autophagy level of MC3T3-E1 cells. The results showed that the osteogenic differentiation and mineralization were reduced, which indicated that autophagy played a crucial role in promoting bone integration by osteoimmune microenvironment.

The limitation of this study lies in the limitation of using MC3T3-E1 cells to study. At the same time, other methods can be used to evaluate the autophagy level, such as transmission electron microscopy, immunofluorescence staining, etc. At the same time, we can further study the autophagy related pathways and mechanisms, and conduct in vivo experiments to further confirm the results of this study.

In this study, RAW264.7 cells were cultured on smooth titanium disks and micro-nano topography titanium disks. It was confirmed that the micro-nano topography titanium disk could stimulate macrophages to differentiate into anti-inflammatory M2 type. The supernatant from the medium was collected to prepare the conditioned medium, which could promote the proliferation, differentiation and mineralization of MC3T3-E1 cells. Autophagy played a key role in this process.

## Methods

### Preparation and characterization of titanium disk

Ti6Al4V, also known as TC4 or Cr5, has been proved to be an excellent implant material for its excellent mechanical properties, biocompatibility and osseointegration^48,49^. In this experiment, Ti6Al4V alloy rod with a diameter of 19.5 mm (Taizhou Yutai metal materials Co., Ltd., Jiangsu) was used to make a 1 mm thick disk, and the surface of the titanium plate was polished to mirror effect. 60 mesh alumina particles (Gongyi Baolai water treatment material factory, Henan) were sprayed on the polished titanium disk surface with a spray angle of 90° and the spraying distance was no more than 5 cm. When the surface of titanium disk had no metallic luster and is uniform gray white, took out the titanium plate, gently blew off the aluminum oxide particles on the surface with a blower, and then immersed the titanium disk in 0.5% hydrofluoric acid solution and treated it at room temperature for 15 min to obtain SLA titanium plate. For alkali-heat treatment, the titanium disk was immersed in 10 mol/L sodium hydroxide solution and treated at 80 °C for 24 h. All titanium disks were placed in 5% concentrated cleaning solution (micro-90, International Products Company, New York, USA), absolute ethanol and three distilled water for ultrasonic vibration cleaning for 5 min respectively, and then they were taken out and placed in the air for natural drying.

For the surface characterization of titanium disk, the surface morphology was observed by cold field emission scanning electron microscope (Carl Zeiss, Germany). The contact angle of the specimen was measured by hanging drop method with 2ul artificial saliva, and the average surface roughness (RA) of titanium disk was measured by Wyko nt9300 optical profilometer (Veeco, USA).

### Cell culture

RAW264.7 cells were provided by Shandong Key Laboratory of oral tissue, and MC3T3-E1 cells were purchased from the National Academy of Sciences cell bank (Shanghai, China). The complete culture medium was α—minimal essential medium (α—MEM, hyclone, USA) with 10% fetal bovine serum (FBS, hyclone, USA), and double antibodies (100 IU/mL penicillin G and 100 μg/mL streptomycin, sorebol, China). The cells were cultured in an incubator with 5% carbon dioxide at 37 °C, and the medium was changed every other day. The control group used osteogenesis induction medium (OM. Hereinafter, the control group was called OM). Osteogenesis induction medium consisted of complete medium supplemented with 50 mg/L of ascorbic acid (sigma Aldrich), 10 mmol/L β—glycerophosphate (sigma Aldrich) and 10 nmol/L dexamethasone (sigma Aldrich). The experiment group used conditioned medium (CM. Hereinafter, the experiment group was called CM). For CM, the supernatant of RAW264.7 cells’ culture medium which was stimulated by micro-nano topography titanium disks, was centrifuged at 1000 g for 5 min, then added with 20% FBS osteogenic induction medium. For autophagy inhibition, 5 mM 3-MA (Shanghai Tao Su Biochemical Technology Co., Ltd., China) was added to the medium to inhibit autophagy.

### Cell counting kit‑8 (cck‑8) detection

Cell proliferation was detected by cell counting kit-8 (CCK-8, dojindo, Tokyo, Japan). MC3T3-E1 cells were treated with osteogenic induction medium or conditioned medium. On the 1st, 3rd, 5th and 7th day, the original medium was replaced according to the ratio of CCK-8 reagent to complete medium of 1:10, and MC3T3-E1 cells were incubated at 37 °C for 1 h, and the absorbance value was detected at 450 nm wavelength with a microplate meter (BMG Labtech, Germany).

### Alizarin Red S staining and determination of cetylpyridinium chloride (CPC)

MC3T3-E1 cells were fixed with 4% paraformaldehyde for 30 min and then incubated with Alizarin Red S (sigma Aldrich) for 10 min. After washing with deionized water, calcium deposition was observed by optical microscope. 10% cetylpyridinium chloride (CPC, sigma Aldrich) was used for quantification, and the absorbance value was detected at 562 nm wavelength with a microplate meter (BMG Labtech, Germany).

### Quantitative real‑time PCR (qRT-PCR)

Total cell RNA was extracted by Trizol reagent (Invitrogen, NY, USA). The cDNA was synthesized using primescript RT Master Mix Kit (Takara biotechnology, China). GAPDH was selected as the internal reference gene, and the relative gene expression ((*runx2*, *collagen I*, *LC3*, *p62*) was calculated by "2^−△ △CT^” method. The sequence of primers was showed in Table 1.

**Table 1:** List of primers used in this study for qRT-PCR.

Genes	Primers	Sequences (5′–3′)
*gapdh*	Forward	AGGTCGGTGTGAACGGATTTG
	Reverse	TGTAGACCATGTAGTTGAGGTCA
*IL-1β*	Forward	GTGTCTTTCCCGTGGACCTT
	Reverse	AATGGGAACGTCACACACCA
*IL-10*	Forward	GCTCTTGCACTACCAAAGCC
	Reverse	CTGCTGATCCTCATGCCAGT
*Runx2*	Forward	GGGACTGTGGTTACCGTCAT
	Reverse	ATAACAGCGGAGGCATTTCG
*collagen I*	Forward	CCCTGGTCCCTCTGGAAATG
	Reverse	GGACCTTTGCCCCCTTCTTT
*LC3*	Forward	CACACCCATCGCTGACATCTA
	Reverse	GAAGGTTTCTTGGGAGGCGTA
*p62*	Forward	CCTCAGCCCTCTAGGCATTG
	Reverse	TTCTGGGGTAGTGGGTGTCA

### Western blot

The cells were lysed with Ripa buffer (wanleibio, China) containing 10 mM protease inhibitor (PMSF; wanleibio, China). The lysate was centrifuged at 12,000 rpm and 4 °C for 10 min. The precipitation was separated and the protein concentration was determined by the BCA protein concentration determination kit (wanleibio, China). The equivalent protein (40 μg) was added to the 8–15% SDS-PAGE gel and then transferred to the PVDF membrane (Millipore, Billerica, USA). The membrane was sealed in 5% skimmed milk powder solution for 1 h, and then incubated overnight at 4 °C with antibodies specific for LC3 (1:500; wl01506; wanleibio, China), runx2 (1:500; wl03358; wanleibio, China), collagen I (1:500; wl0088; wanleibio, China), β-actin (1:1000; wl01845; wanleibio, China). After washing 4 times with TBST, the membrane was incubated with goat anti-rabbit IgG-HRP (1:5000; WLA023; wanleibio, China) for 45 min at room temperature. The protein bands were visualized using ECL detection kit (wanleibio, China) and compared on the same membrane. We used gel image processing system (Gel-Pro-Analyzer software) to analyze the optical density of target strips and calculate the proportion of LC3-II/I in order to determine the level of autophagy.

### MDC staining

MDC (Leagene, China) staining was used to determine autophagy activity^50^. In short, the cells were washed in Hanks balanced salt solution (HbSS) and treated with 10 μM MDC (in HbSS) at 37 °C for 30 min. Cells were rinsed with PBS four times and imaged by inverted fluorescence microscope (Olympus IX71, Japan).

### Statistical analysis

The quantitative results were expressed as mean ± standard deviation (SD). The experiment was repeated at least 3 times independently. One-way ANOVA and multiple T test were used for statistical evaluation, p < 0.05 was considered to be statistically significant.

## Supplementary Information

Supplementary Information 1.

## Abbreviations

SLA: Sandblasted, large grit, acid-etched
OM: Osteogenesis induction medium
CM: Conditional medium (osteogenic induction medium and the supernatant of RAW264.7 cells’ culture medium)
CCK-8: Cell counting kit-8
qRT-PCR: Real-time quantitative reverse transcription PCR
RA: Average surface roughness
SEM: Scanning electron microscope
MDC: Monodansylcadaverine
