# Supplementary figures and images for: The role of autophagy in the process of osseointegration around titanium implants with micro-nano topography promoted by osteoimmunity
Source: Sci Rep. 2021 Sep 16;11:18418. doi: 10.1038/s41598-021-98007-7 (PMC8446058; doi:10.1038/s41598-021-98007-7)

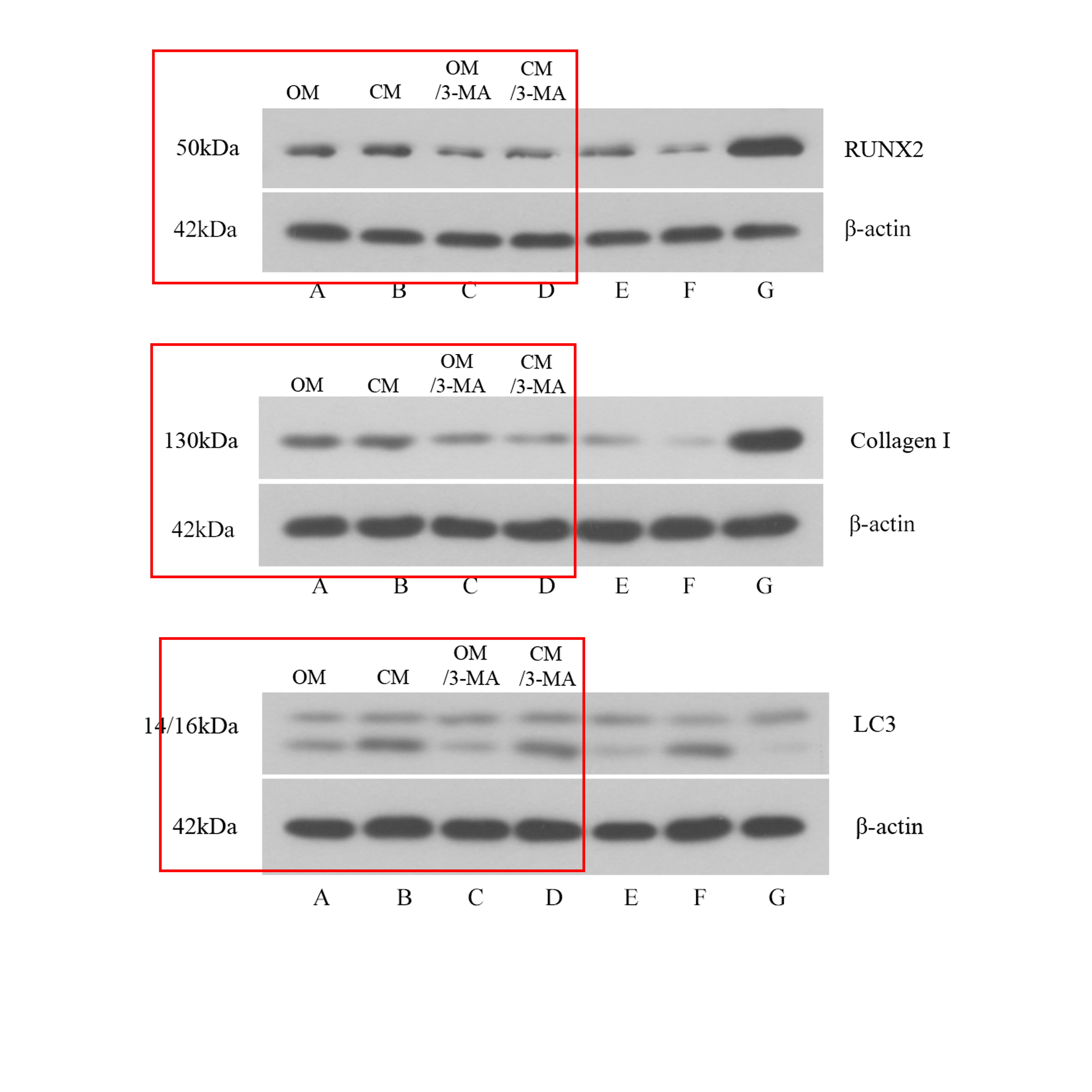

Supplement: Supplementary file 1 — Supplementary Information 1. [file 41598_2021_98007_MOESM1_ESM.tif]
